# Perception of Minimum Interventional Dentistry among Dental Undergraduate Students and Interns

**DOI:** 10.3390/medicina59040649

**Published:** 2023-03-24

**Authors:** Manal M. Abdelhafeez, Fatima M. Alharbi, Swati Srivastava, Elzahraa Eldwakhly, Selma A. Saadaldin, Mai Soliman

**Affiliations:** 1 Department of Conservative Dental Sciences, College of Dentistry, Qassim University, P.O. Box 1162, Buraydah 51452, Saudi Arabia; 2 Department of Endodontics, Faculty of Dentistry, October University for Modern Sciences and Arts, 6th of October City 12451, Egypt; 3 Department of Prosthodontics, College of Dentistry, Qassim University, P.O. Box 1162, Buraydah 51452, Saudi Arabia; 4 Department of Clinical Dental Sciences, College of Dentistry, Princess Nourah bint Abdulrahman University, P.O. Box 84428, Riyadh 11671, Saudi Arabia; 5 Prosthodontics Division, Schulich School of Medicine and Dentistry, Western University, London, ON N6A 5C1, Canada

**Keywords:** minimum interventional dentistry, knowledge, attitude, practice, dental students, interns

## Abstract

*Background*: The philosophy of minimum interventional dentistry (MID) is to integrate prevention, remineralization, and minimal intervention for the placement and replacement of restorations. All branches of dentistry play an important role in practicing MID, and their primary goal is to realize that any restoration is of less biological significance than the healthy original tissue Objectives: The objective of this study was to assess the perception of MID among dental undergraduate students and interns in terms of knowledge, attitude, and practice at the College of Dentistry. *Materials and Methods*: This cross-sectional study was conducted among undergraduate students and interns at the College of Dentistry, Qassim University, Saudi Arabia. A self-administered questionnaire was distributed, which included basic demographic profiles and questions about the knowledge, attitude, and practices toward MID. The data were tabulated in MS Excel, and all statistical analyses were performed using SPSS version 21. *Results*: A total of 163 dental students were recruited, with senior students comprising 73% and interns comprising 27%. Male students were slightly more prevalent (50.9%) than female students (49.1%). About 37.6% of participants received training about MID during educational courses, while 10.3% received it during their internship. A statistical test revealed that the prevalence of interns who were trained in performing MID was significantly higher (*p* < 0.001). *Conclusions*: The majority of the participants demonstrated proper knowledge, attitude, and practice in different aspects of MID. Interns reported a higher rate of knowledge, attitude, and practice in MID compared to undergraduate students. However, more education and hands-on training about MID concepts during the college curriculum are necessary to attain better knowledge, attitude, and practices that could be useful for more conservative clinical practice.

## 1. Introduction

The philosophy of minimum interventional dentistry (MID) is to integrate prevention, remineralization, and minimal intervention for the placement and replacement of restorations to preserve tissue by preventing disease and intercepting its progress with as little tissue loss as possible. All branches of dentistry play an important role in practicing MID, and their primary goal should be to realize that any restoration is of less biological significance than the healthy original tissue [1].

Minimally invasive restorative dentistry is a recent approach used to describe more conservative tooth preparation and restoration in operative dental practice [2,3,4,5]. It includes risk assessment of caries and patients’ reinforcement for self-care, early detection before cavitation begins, pit and fissure sealants in unaffected zones, minimal surgical intervention of carious lesions, and fluoride application. Many techniques are employed, including hand instrumentation, chemo-mechanical caries removal, air abrasion, and laser cavity preparation, starting from diagnosis to treatment and early detection of any demineralized tooth structure. Adequate knowledge is necessary to adopt the current advanced method [6,7,8,9,10,11,12].

The “golden triangle” of minimally invasive operative caries management considers the three factors of tissue histology, dental biomaterials science, and clinical handling of the patient and materials to permit the successful implementation of minimally invasive dentistry in all patients [13].

Glass ionomer cement (GIC) is one of the most famous remineralizing biocompatible agents that have a major role in MID. It was developed in England in 1972 by Wilson and Kent [14] and entered the field of dentistry by 1988 [15]. GIC specifically attracts to enamel and dentin through a specific chemical bond that results in the formation of a unique acid-resistant interface, ensuring a highly technical-tolerated and long-lasting restoration [16], in addition to its superior property of fluoride release and continued remineralization [17,18,19].

Minimum intervention oral healthcare (MIOC) is a team-delivered, patient-focused oral health delivery framework applicable to any of the restorative disciplines (including cariology) and crosses all patient demographics, with suitable modification. It involves four interlinked domains of care: identifying problems, prevention and control, minimally invasive dentistry (MID), and review and recall [13,20,21].

The International Caries Classification and Management System (ICCMS^TM^) is a comprehensive set of clinical protocols that aims to preserve tooth structure and restore only when necessary [22]. Based on critical analysis, research, and clinical feedback, the ICCMS^TM^ emphasizes prevention and the restoration of moderate or extensive caries lesions while preserving as much natural tooth structure as possible [23]. The ICCMS^TM^ is implemented through the International Caries Detection and Assessment System (ICDAS^TM^) and is consistent with the World Dental Federation’s (FDI) policy on caries classification and management [24].

A thorough understanding of the clinical relevance of contemporary adhesive dental materials science is required to successfully implement the MIOC. The physicochemical interaction of the relevant dental substrate retained at the cavity surface with the adhesive material must be enhanced by the operator to achieve medium- to long-term successful outcomes [25].

On the other hand, and moving to the endodontic branch of dental science, within the scope of “Minimally Invasive Endodontics”, the procedures can range anywhere, starting from endodontic diagnosis and decision of endodontic intervention, access opening with minimal dentin removal based on canal anatomy, preparation of canals to retain maximum sound dentin to deciding the nonsurgical or surgical retreatment procedure of choice and moving to the concept of ninja access in endodontics, with all of its benefits and drawbacks, that was basically introduced as a recent attitude to perform a very small and tiny access preparation to complete root canal treatment using micro instruments, with the aid of suitable illumination and magnification equipment as well [26,27]. The dentist must develop the required skills and manual dexterity to perform endodontic procedures in a limited working area specific to the treatment of the cause [28,29,30,31]. In aid, appropriate imaging modalities, such as cone beam computed tomography, micro-computed tomography, and magnifications as well, should be utilized for better understanding and visualization of the root canal system whenever indicated [32].

The recent paradigm shift in fixed prosthodontics has been significantly influenced by current advancements in adhesive sciences and implant dentistry [33,34]. These changes have dramatically altered the way practitioners diagnose, treatment plan, and perform clinical dentistry by driving clinicians to think in terms of conserving tooth structure, vital tissues, and aesthetics. Traditional restorative concepts that advocated the removal of critical tooth structures have been modified to promote the preservation of those same tooth structures [19,20]. Similarly, laminate veneers, all-ceramic occlusal onlays, and resin-bonded fixed partial dentures have made it possible to preserve the natural tooth structure of the potential abutment teeth. Hence, a less invasive preparation and restoration design appear to have a favorable effect on the vitality of restored teeth. Against this background, fixed prosthodontics has been undergoing a paradigm shift towards less invasive treatment methods in recent years [35,36].

Minimally invasive restorations are beneficial because they reduce the risk of devitalization, are kind to the tooth structure, and offer high esthetic potential. Whilst these possibilities inspire a great deal of euphoria, prosthodontists should bear in mind that minimally invasive restorations involve a high degree of technique sensitivity concerning preparation (mainly in the enamel), adhesive bonding, and final fine-tuning of the static and dynamic occlusion [37,38]. Adhering to the defined guidelines of conservatism during the various clinical and technical treatment phases presents a key factor for achieving clinical long-term success.

As MID has evolved during recent decades, many of the traditional principles, operative technologies, and the restorative materials taught in dental school curricula in the past have all developed and changed significantly. Contemporary training requires an alternative modern-day skillset to be appreciated fully and used effectively in the correct clinical circumstances. Patient attitudes have also changed in terms of their expectations of modern dental care and the desired outcomes with respect to the management of dental disease. Dental professionals can, therefore, no longer rest on their laurels and cannot afford to rely on outdated principles and techniques. They must embrace these evolutions in ideologies, technologies, and materials, as has occurred in other aspects of general healthcare and even society in general [39].

For MID to be truly embedded as the underpinning care delivery format, it must be taught and promoted both at undergraduate and postgraduate levels. In conjunction with communication/interviewing skills and appropriate medicolegal documentation, minimum intervention care is relevant to all specialties of dentistry, not just caries management in conservative dentistry [39].

Furthermore, it is the responsibility of dental schools to equip future dentists and members of the oral healthcare team with the core minimum intervention skills, competencies, and understanding to be able to care for the patients of the future, whose needs will be different from those of the past. This requires new as well as established dentists to understand the changes in oral healthcare perceptions from both the patients’ and professionals’ point of view. These changes must be reflected and applied to modern, unified under/postgraduate dental curricula. Contemporary learning outcomes accompanied by rigorous longitudinal assessment are in need of development rather than relying solely on the traditional educational outcome formulas and examination of the past [39].

Currently, there are a proportion of practitioners working who may not have the confidence required to practice the appropriate skillsets for MID optimally and who need good quality postgraduate education and continuing professional development. This can be delivered in a variety of modes, including lectures, seminars, and hands-on courses. However, the real value of practical take-home information gleaned from many of these courses is debatable and it is impossible to assess or verify their implementation [39].

The treatment a dentist adopts is most likely a result of the dental school training practice experience and the continuing dental education [40]. Studies have reported that the dental schools must accept the responsibility for teaching more important and complex aspects of care, such as practice to arrest initial caries progression and prevent the formation of cavity rather than to focus on surgical intervention, as the prime measure to control caries [41]. These approaches have been incorporated in the structure of various dental curricula worldwide [2] so that, while passing through the professional educational curriculum, each dental student has adequate professional knowledge, skill, beliefs, and attitude.

Despite emerging evidence on effectiveness of preventive caries protocols, the dental curriculums especially in Europe and Australia have undergone a paradigm shift towards teaching the medical model of caries management, based on caries risk assessment and minimal invasive strategies to treat dental caries [42,43]. In contrast, dental schools in the Middle East and Asia still follow the traditional Greene Vardiman Black (G.V. Black) classification at the undergraduate level, which is based on the principles of cavity designs. This influences their clinical outlook, especially contemporary caries management principles because the same students become future clinicians. They continue performing what they learned in their dental school [44].

The outcome of the studies conducted in Saudi Arabia regarding the practice of MID principles has revealed a lack of knowledge and implementation of MID principles [45,46]. Although, most of these studies involved general dental practitioners who belong to different nationalities, educational backgrounds, and follow different schools of thought and, thus, vary in their outlook and practices. The conventional practice of caries management not only has a biological cost, but financial burden also, as most of the time and effort in general dental practices are spent on restoration, repair, and replacement of existing failing restorations [47].

Several studies revealed scarce data on the outcome of MID practices within Saudi Arabia. Out of these studies conducted, the majority have inculcated general dentists practicing in Kingdom. Since there are many schools of thought involved, their way of practice differs from each other too [48,49,50].

Among the range of core competencies necessary for contemporary practice in oral healthcare, patient-centered care (PCC) and minimally invasive dentistry (MID) are central for positive patient health outcomes [51,52,53]. Therefore, our curricula need to ensure our students have developed these competences on graduation.

College of Dentistry Qassim University adopted a radical shift in the dental curriculum towards MID, which is now an inbuilt component of the present undergraduate dental curriculum. It is taught from third year to fifth year, which focusses mainly on clinical branches of dentistry. The undergraduates are given didactic training of MID essential in tooth preservation across various branches of dentistry, such as operative dentistry, endodontics, and prosthodontics. However, whether this knowledge of MID is practiced effectively by the students later when they start their dental practice is still doubtful and of concern.

Therefore, as dental academics, we need to identify research questions of importance to our students’ learning and, ultimately, their patients’ health outcomes and then ensure our research designs enable us to answer these questions. In turn, we need to report the outcomes appropriately.

Despite MID being a treatment philosophy that emphasizes protection of existing tooth structure and it has been incorporated in the dental curricula worldwide in comprehensive patient care, there is limited evidence whether the familiarity with MID principles imbibed through the curriculum is translated into clinical decision making and practice.

This study assesses the perception of the dental undergraduate students and interns of Qassim University, Saudi Arabia, to comprehend the notion of MID in their learning. Hence, this study aimed to assess the knowledge of undergraduate students and interns about MID principles and techniques and to clarify whether a positive attitude is reflected after gaining knowledge and concepts of MID.

## 2. Materials and Methods

This cross-sectional study was conducted to assess the knowledge, attitude, and practice of minimum interventional dentistry among undergraduate students (third-, fourth-, and fifth-year students) and interns (students in the internship year (12 months) to meet a mandatory requirement for full registration as Dental Practitioner) in College of Dentistry, Qassim University. Ethical approval was obtained from Dental Ethical committee, Dental Research Center, College of Dentistry, Qassim University (Code #: EA/F-2019-3014). A questionnaire was adapted from several previous studies [47,48,54,55,56] and new questions were originally created to include questions testing application of MID in Endodontics and Prosthodontics prospective. For validation, four arbitrators from the College of Dentistry, Qassim University, self-assessed and evaluated the questionnaire. The questionnaire was modified according to their inputs. The questionnaire consisted of two parts; the first part gathered basic demographic profiles, academic year level and gender, and one question for the time the participant received MID training. The second section of the questionnaire was divided into two subsections; the first subsection assessed the knowledge, with nine closed-ended multiple-choice questions (MCQs) based on a Likert scale ranging from strongly disagree (1) to strongly agree (5), and the second subsection assessed the attitude and practice, with nine closed-ended MCQs ranging from never (1) to always (4).

The survey targeted 227 undergraduate students and interns in College of Dentistry, Qassim University, Saudi Arabia. The questionnaire was converted to an online electronic form using Google Forms (Google Forms, 2019; a free web-based survey generator). A link to the questionnaire was generated and distributed at the end of the academic year 2020–2021 twice via official emails. The collected data were subjected to descriptive and statistical analysis.

### Statistical Analysis

Descriptive statistics had been presented using numbers and percentages. Dental undergraduate students and interns were compared regarding the knowledge, attitude, and practices of MID concepts by using Fischer Exact test; *p* < 0.05 was considered statistically significant. A chi-square test of independence as well as a Monte Carlo analysis were performed. Data analysis was performed using Statistical Packages for Software Sciences (SPSS) version 21 (Armonk, IBM Corporation, New York, NY, USA).

## 3. Results

**Figure 1:**
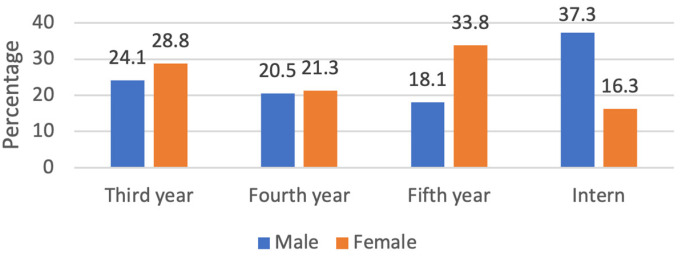
Respondents ‘distribution according to students’ academic year level in relation to gender.

**Figure 2:**
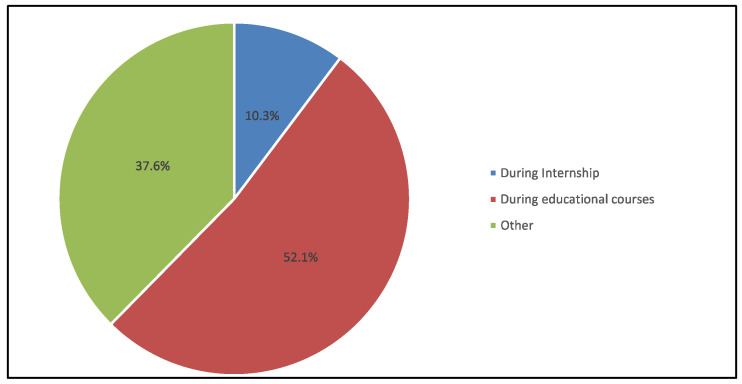
Respondents’ responses to the time they received training for MID.

In total, 163 responses were recruited out of 227, with a response rate of 71.8% (undergraduate students: 73% vs. interns: 27%). Males’ response of 83 (50.9%) was slightly greater than females’ response, which was 80 (49.1%). The most common academic year level was interns, which recorded 44 (27%), followed by third-year level at 43 (26.4%) and fifth-year level at 42 (25.8%), while the least of them was the fourth-year level at 34 (20.9%) (Table 1). Furthermore, it was observed that there were more male intern respondents than female interns (37.3% vs. 16.3%), whereas there were more females in the fifth-year level than males (33.8% vs. 18.1%) (Figure 1). It was observed that more than half of participants (52.1%) received the training during their educational courses and 10.3% received their training during the internship (Figure 2).

Nearly half of the respondents declared that they have received training for MID during the educational courses (52.1%), while 10.3% of the respondents have received MID training during their internship (Figure 2).

Nearly 90% of the respondents agreed that dietary habits should be evaluated in all patients. Furthermore, the agreement that Caries Risk Assessment (CRA) should be performed with all patients was relatively high, as more than 89% agreed or strongly agreed about it. They also highly agreed (80%) that conservative cavity designs, such as tunnel and box preparation, are effective for tunnel restoration. However, only about 60% of the respondents reported that the use of lasers for caries detection can be frequently applied (Table 2).

In total, 86% of the respondents agreed or strongly agreed that access openings must be crafted to preserve sound tooth structure. Around 31.8% of the respondents were either uncertain or disagreed on the use of Gates Glidden drills on a routine basis during access cavity preparation. Most of the respondents (93.87%) were uncertain or disagreed on reduction of bacterial load from the canal by preparing large apical size (Table 2).

Furthermore, a majority of the respondents (89.57%) believed that conservative preparation designs should be applied to receive extra coronal restoration. Moreover, 82.7% of the respondents agreed that the improvement of the recent adhesive system resulted in increasing the success rate of conservative preparation designs (Table 2).

Pertaining to their attitude and practices, a majority of the respondents exhibited positive attitudes and practices toward the MID concepts and tools. For example, 47.2% and 44.8% of the respondents always or often apply the concepts concerning the planning of the used restorative materials and techniques based on the patient’s CRA. However, the familiarity toward the use of magnification (e.g., loupes) was poor; 9.2% and 11.6% of the respondents stated that it should be used “sometimes” or “often” and only 19.6% believe that it should be used “always” (Table 3).

Responses regarding familiarity of using recent high-strength and high-esthetic ceramic materials revealed that about half of the participants are “Always” and “Often” (17.18% and 31.90%) familiar with using such materials in their practice. However, about 42% of the participants responded by “Always” and “often” regarding familiarity with using different bonding systems and techniques for bonding to different ceramic restorations. Meanwhile, about 17% of the participants responded by “Never” being familiar with different bonding systems and techniques (Table 3).

More than half of the participants “Sometimes” and “Never” experience using different bonding techniques for cementation of all-ceramic restorations (Table 3).

A chi-square test of independence as well as a Monte Carlo analysis were performed to examine the relation between dental undergraduate students and interns and their knowledge toward the importance of the patient’s dietary habit and caries risk assessment. The relation between these variables was significant. The interns were more likely to have knowledge in these questions than undergraduate students. Concerning the knowledge toward the various conservative cavity designs as well as the use of lasers for caries detection, the relation between these variables was also significant. The interns scored more knowledge achievement rather than students (*p* < 0.001) (Table 4).

Going through the knowledge about the minimum invasive endodontics, whether through the concepts of crafting the access cavity or the routine use of Gates Glidden during canal preparation and allowance of large apical sized preparations as a way to reduce the bacterial loading inside the prepared canal, it was found that the interns more often stick to the principles of minimum invasive endodontics rather than the undergraduate students, with a significant difference between them (*p* < 0.001) (Table 4).

Regarding knowledge toward the fixed prosthodontics principles in minimum interventional dentistry, it was found that the interns’ knowledge about conservative preparation designs and of recent adhesive systems exceeded that of the undergraduate students in general, with a significant difference (*p* < 0.001) between both groups (*p* < 0.001) (Table 4).

After finalizing the whole data and giving scores to the responses of both groups, we found that there is a significant difference in the scoring value of knowledge statement between them and that the interns’ scoring of knowledge toward minimal invasive dentistry scoring was significantly higher than that concerning the undergraduate students (Table 5).

A chi-square test of independence as well as a Monte Carlo analysis were performed to examine the relation between undergraduate students and interns and their attitude and practice toward planning the use of restorative materials and techniques according to the patient’s caries risk assessment, the use of magnification tools for tooth preparation or radiographs for caries detection, and the use as well of chemo-mechanical cavity preparation techniques in the clinics. The final relation between these variables was significant (*p* < 0.001). The interns were more likely to practice the concepts and tools for minimum interventional dentistry rather than undergraduate students (Table 6).

However, concerning the practice of minimal invasive endodontics, specifically through the preparation of minimal-sized access, it was found that there was no significant difference (*p* = 0.064) between the attitude and practicing of both undergraduate students and interns in applying the principles of minimum invasive endodontics, as most of them are trying to keep this concept along while practicing endodontic treatment (Table 6).

A significant difference in the attitude and practice of using Gates Glidden drills was seen between undergraduate students and interns. A total of 100% of interns often or always used these drills regularly during biomechanical preparation as compared to only 40.3% of undergraduate students (Table 6).

Furthermore, 90.9% of interns agreed that they never practiced the concept of larger apical size preparation to decrease the bacterial load. This was significantly different from 87.4% of undergraduate students who “sometimes” or “often” or “always” practiced preparing larger apical sizes to reduce the bacterial load inside the canal (Table 6).

Going through the use of recent high-strength and high-esthetic ceramic materials as well as the use of different bonding systems and techniques for bonding to different ceramic restorations, it was found that the interns are practicing the prosthodontic principles of minimum interventional dentistry at a higher prevalence and with a significant difference (*p* < 0.001) than the undergraduate students (Table 6).

On finalizing the total available data and giving scores to the responses of both groups, we also realized that there is a significant difference in the scoring value of attitude and practice between them, and that the interns’ scoring of attitude and practice toward minimal invasive dentistry was significantly higher than that concerning the undergraduate students (Table 7).

## 4. Discussion

This study sought to determine the knowledge, attitude, and practices of dental undergraduate students and interns regarding minimum interventional dentistry (MID) concepts in the College of Dentistry, Qassim University, Saudi Arabia.

In the current study, 90% of the respondents agreed that dietary habits should be evaluated in all patients. Furthermore, the agreement that Caries Risk Assessment (CRA) should be performed with all patients was relatively high, as more than 89% agreed or strongly agreed about it. They also highly agreed (80%) that conservative cavity designs, such as tunnel and box preparation, are effective for tunnel restoration. However, there were only about 60% of the respondents who reported that the use of lasers for caries detection can be frequently applied.

Concurring results were presented by Alrasheedi H. et al., (2020) [47], who reported that 78.9% participants agreed about the importance of performing caries risk assessment (CRA) for all patients. Likewise, 87% of the participants would plan restorative treatment with materials and techniques based on individual caries risk assessment. These findings are in unison with the policies of American Academy of Pediatric Dentistry (AAPD) [57] and reflected a positive attitude towards evidence-based learning. The findings are in contrast to a similar study conducted in Jaipur, India, by Nagaraj A. et al., (2015) [58], wherein only one fourth of the study participants practiced CRA out of the 80% who were aware of it.

The importance of noninvasive techniques, such as CRA and use of pit and fissure sealants in caries control, needs to be strictly reinforced in undergraduate learning and clinical practice according to the American Dental Association (ADA) recommendations [59].

In addition, similar results were presented by Alrasheedi H. et al., (2020) [47], as they found that 33.2% of their respondents strongly agreed and 41.4% agreed that conservative cavity design, such as tunnel and box preparation, is effective. However, 10.7% collectively were uncertain about the role of carbohydrates in etiology of dental caries and, further, 25% expressed their disagreement with the effectiveness of conservative cavity designs, such as tunnel preparations and box. Moreover, they reported a comparative result that use of magnification and contemporary caries detection tools, such as electric caries monitor (ECM), quantitative light-induced fluorescence (QLF), infrared laser fluorescence (IRLF), and fiber-optic trans-illumination (FOTI), was not applied by 51% and 50% of participants, respectively. The collective response of the dental students towards techniques for MID practice revealed that the majority 91% of respondents consider the use of conservative restorative techniques, such as the sandwich technique and atraumatic restorative technique (ART), 86% as “effective“ over the conventional restorative methods [47].

A recent systematic review concluded that laser caries removal is not yet a viable general dental practice option for effective caries excavation [25]. Enzymatic (including hypochlorite-, pepsin-, and papain-based) solutions have and are being investigated to help further breakdown of collagen in already softened carious dentine in the hope of developing a more self-limiting technique of removing caries-infected dentine alone.

Bhatiya P. et al., (2015) [60] reported that CRA should be performed for all the patients, since it proves to be an effective way for dental caries assessment. In a study by Natarajan and Prabakar [54], they documented that the knowledge of dental professionals regarding MID was found to be high, which was consistent with our study.

Furthermore, a modest level of knowledge of the noninvasive method of caries management was observed in the fourth-year Australian dental students [41]. These results were similar to the study of Brazilian dental professionals (with <5 years after graduation), where the majority had adequate knowledge about MID procedures [4].

Students’ attitude toward caries prevention can have a bearing on their training and consequently the approach toward preventive services that they are likely to provide in their future practices [61].

Another study conducted by Agrawal R et al., (2014) [62] reported an overall positive attitude toward MID by most of the interns, such as application of pit and fissure sealant. Similarly, the attitude of dental interns in this study toward caries risk assessment was positive, whereas, in the other study on dental interns in India, 49.66% reported that they would use caries activity test in the future [62]. About 90% of the dental interns agreed that fluoride application was an effective way of preventing caries, similar to the responses of dental interns in the other Indian study and also among Iranian senior dental students [62,63], while most of the third- and fourth-year dental students in Florida were willing to monitor and arrest enamel lesions in their practices [64].

On the other hand, in a Brazilian study [65], researchers revealed conflicting reports. They documented that, although most of the respondents were aware of the MID procedure, 49.6% of them did not follow it in their daily practice and a similar proportion (48.4%) did not believe in the technique or they did not have enough knowledge on how to perform it.

In this study, we found that around 86% of the respondents agreed or strongly agreed that access openings must be crafted to preserve sound tooth structure. This is especially critical to avoid gouging cervically, laterally, or into the floor of the pulp chamber [29].

Results of the current study revealed that around 59.5% of the respondents have never utilized magnification for tooth preparation and caries detection. Only 19.6% of the respondents were always using magnification tools during operative procedures. Use of radiography is still used solely for caries detection, irrespective of the training given for MID. Identification of incipient lesions by using magnification should be implemented and used during operative procedures on a routine basis [66].

Around 31.8% of the respondents were either uncertain or disagreed on the use of Gates Glidden drills on a routine basis during access cavity preparation. Gutmann has stated that, while judicious orifice location and careful canal penetration are essential, efforts should be made to minimize the excess removal of cervical tooth structure in the canal orifice through the use of Peeso reamers, Gates Glidden drills, and orifice opening instruments. The literature indicates that loss of tooth structure cervically weakens the tooth and makes it susceptible to fracture [67,68].

Furthermore, 93.87% of the respondents were uncertain or disagreed on reduction of bacterial load from the canal by preparing large apical size. While the concept of larger apical sizes has received some literature credibility with regards to bacterial reduction, maintaining smaller sizes when possible (>#20 or ≤#40) would seem desirable for preservation of radicular dentin in the majority of cases. It would then seem reasonable to develop better methods of canal cleaning and disinfection that can be used in the presence of retained, sound tooth structure [29].

There was a significant difference in the knowledge of undergraduate students and interns, as interns adhered to the principles of minimal invasive endodontics as discussed above significantly more than the dental students. This might be attributed to their increased skills, knowledge, and experience in the field.

Most of the respondents (89.57%) believed that conservative preparation designs should be applied to receive extra coronal restoration. Moreover, 82.7% of the respondents agreed that the improvement of the recent adhesive system resulted in increasing the success rate of conservative preparation designs.

A study conducted by Brunton et al., (2005) [69] revealed that laminate veneers were preferred by younger practitioners. Despite porcelain fused to metal crowns requiring a deeper preparation, which can have adverse consequences on the pulpal–dentinal complex, leading to a loss of vitality in around 19% of crowned teeth, the preservative potential of minimum invasive techniques, such as resin-bonded crowns, is accepted but there will be cases where porcelain fused to metal crowns are still required [69].

Jum’ah et al., (2019) [70] found that there was a notable increase in the percentage of practitioners prescribing direct resin composite veneers in comparison to the 2008 survey [71]. This indicates that the application of minimally invasive, additive, and retrievable treatment concepts was improved.

Limiting tooth structure removal to the extent that it fulfils optimum mechanical and aesthetic requirements is paramount. Using pre-preparation matrices constructed on a waxed-up model is one of the most efficient means to control tooth structure removal. It also enables clinicians to perform “smart” preparations, especially in the cases of misaligned and/or worn teeth. Moreover, depth cutting burs could be used for more conservative preparation [69].

It is suggested that improved adhesives, esthetics, and mechanical properties of resin composite systems are considered to have given practitioners confidence in delivering life-like, predictable restorations. Reinforcing such trends should be a priority in both undergraduate and postgraduate programs [70].

Regarding fixed prosthodontics knowledge principles in minimum interventional dentistry, it was found that there is a significant difference (*p* < 0.001) between the undergraduate students’ and interns’ knowledge about conservative preparation designs and different adhesive systems. This might be attributed to the improved knowledge, skills, and experience of the interns in the field compared to undergraduate students.

Pertaining to attitude and practices, most of the respondents exhibited positive attitudes and practices toward the MID concepts and tools. For example, 47.2% and 44.8% of the respondents “always” or “often” apply the concepts concerning the planning of the used restorative materials and techniques based on the patient’s CRA. However, the familiarity toward the use of magnification (e.g., loupes) was poor; 9.2% and 11.6% of the respondents stated that it should be used “sometimes” or “often” and only 19.6% believed that it should be used “always”. Conversely, the use of radiographs for the detection of caries was well practiced by the respondents, since many of them do not suggest or are not accustomed to the use of either the recently innovated caries detection tools or the chemo-mechanical agents for caries removal with minimal destructive preparations [72,73,74,75]. That seems to go in accordance with their maximum dependability on specific radiological assessments for caries detection [76,77,78].

The poor utilization of magnification loupes and the lack of appreciation in the newer methods were consistently reported in the literature [48,56,57,59,79,80]. Alrasheedi et al. [47] also pointed out that there was a scarcity of applications towards contemporary caries detection methods, as most of the participants still followed traditional caries diagnosis. They further expounded that the use of magnification and contemporary caries detection tools were not applied by half of the participants, which was consistent with our results. Although it is well-accepted fact that use of magnification is an inherent aspect of contemporary dentistry and significantly enhances diagnostic ability [81], the cost and unavailability of these noninvasive contemporary diagnostic aids could be a factor of their limited use.

Regarding attitude and practice for minimal invasive endodontics, no significant differences were found in the practice of minimum-access cavity preparation in general between undergraduate students and interns. It was evident that all of them adhered to the practice of minimal tooth cutting during access preparation and were directed to maximum tooth conservation irrespective of significant difference in their knowledge. This might be attributed to their academic training and experience.

On the other hand, a significant difference in the attitude and practice of using Gates Glidden drills was seen between undergraduate students and interns. In total, 100% of interns often or always used these drills regularly during biomechanical preparation as compared to only 40.3% of undergraduate students. This significant difference might be attributed to increased practice of rotary systems in endodontics by the interns as well as their superior control over their instruments that they gained from their experience and that mostly allowed them to avoid the hazards and the misuse of either the Gates Glidden or the rotary filing systems as well. The use of Gates Glidden drills deep into the root canal should be abandoned in favor of minimally tapered rotary instruments (no larger than 0.06). The former instruments tend to straighten the canal, weaken the root walls and predisposing them to cracks, and, in some cases, leads to irreparable defects, such as root wall stripping defects, especially when we are discussing the preparation of curved root canals mostly in molar teeth. However, these drawbacks and limitations are greatly reduced in cases of straight root canal preparations as in anterior teeth [29].

Furthermore, 90.9% of interns agreed that they never practiced the concept of larger apical size preparation to decrease the bacterial load. This was significantly different from 87.4% of undergraduate students who “sometimes” or “often” or “always” practiced preparing larger apical sizes to reduce the bacterial load inside the canal. Studies have shown that minimal sizes can accomplish this task of elimination of bacteria as adequately as larger diameters [82,83,84].

Responses regarding familiarity of using recent high-strength and high-esthetic ceramic materials revealed that about half of the participants are “Always” and “Often” (17.18% and 31.90%) familiar with using such materials in their practice. However, about 42% of the participants responded by “Always” and “often” regarding familiarity with using different bonding systems and techniques for bonding to different ceramic restorations, while about 17% of the participants responded by “Never” being familiar with different bonding systems and techniques.

Jum’ah et al., (2019) stated that the percentages of dental practitioners who prescribe metal-free crowns has almost doubled since 2008. This might be due to increased patient expectations and demands for metal-free restorations [70]. Additionally, advances in dental biomaterials sciences have allowed high-strength ceramics to be produced that can withstand occlusal forces and produce optimum esthetic results. Lithium disilicate and zirconia-based ceramics were considered among the most trustworthy materials to construct metal-free crowns, inlays, and onlays by most of the respondents [70].

In a survey conducted by K. Klosa et al., (2016), they found high rates of improper bonding techniques used for adhesive cementation of all-ceramic restorations. However, there was an improvement in the values of evidence-based bonding procedures for oxide ceramics recorded, while that for silicate ceramics declined during the observation period of the study [85].

The results of a survey conducted by Rauch et al., (2020) suggested that dentists in Germany select the cementation regime depending on the restorative material. Some of the participating dentists selected cementation regimens that are less or not at all recommended for restorations fabricated from zirconia-reinforced lithium-silicate glass ceramic and CAD/CAM resin composites or were not sure about the appropriate cementation protocol [86].

In the current study, it was reported that more than half of the participants “Sometimes” and “Never” use different bonding techniques for cementation of all-ceramic restorations. This might not be due to a lack of knowledge of bonding systems but rather as a result of additional expenses of air-abrasion devices and different types of silane and primers [87].

Regarding fixed prosthodontics attitude and practice in minimum interventional dentistry, there was a significant difference (*p* < 0.001) between the undergraduate students’ and interns’ attitude and practice regarding recent high-strength and high-esthetic ceramic materials, as well as the use of different bonding systems and techniques for bonding to different ceramic restorations. Interns recorded a higher rate of knowledge, attitude, and practice in minimum interventional dentistry exceeding those of the undergraduate students in general, which might be attributed to their increased skills, knowledge, and experience in the field.

Gaurav Gupta et al., (2014) conducted a cross-sectional survey among dental interns of all the dental colleges in Bengaluru city, and they found that the mean scores for knowledge (3.40 ± 0.85), attitude (18.74 ± 2.8), practice (27.55 ± 6.8), and behavior (1.11 ± 0.9) showed that these interns had adequate knowledge and a positive attitude toward MID. However, their behavior toward MID was negative, and they did not practice MID very often. The mean attitude score showed a significant correlation with knowledge and practice. They concluded that interns exhibited adequate knowledge and positive attitude, which they acquired through their undergraduate curriculum, but it failed to create positive behavior toward practicing MID. Hence, it can be suggested that there is a need to instill positive behavior among students so that they practice MID routinely [88].

Although the aim of the undergraduate program was for MID to underpin the entire five years, its implementation was limited for various reasons. Despite being exposed to many hours of scenarios, tutorials, seminars, and class meetings, students did not apply MID as well as expected in the clinic based on tutor feedback. It appeared that students lacked the skills to implement what they had learned, but the reasons for this are difficult to identify [43].

Educators often assume that knowledge transfer will occur from the classroom to the clinic, but this is not always the case. Students may struggle to obtain essential patient information necessary to develop a tailor-made management plan, hindering their ability to transfer their knowledge into the clinic. In addition, other factors, such as quotas set by clinical disciplines, inconsistent treatment planning, and managing difficult patients, made holistic patient management challenging for students [43].

The attitudes of faculty members and institutional policies also have a significant influence on how students choose materials or concepts for their future practices. To improve MID implementation, all staff from different disciplines must engage with the process and be willing to work together rather than in isolation, as often happens in dental schools with separate departments [43].

A strength of the current study is that it investigated MID knowledge, attitude, and practice across multiple disciplines, including restorative dentistry, endodontics, and fixed prosthodontics. However, the limited number of participants may have adversely affected the study’s generalizability. To address this, a second study should broaden the target population to include undergraduate students and interns from multiple dental colleges in Saudi Arabia. A third study could cover further dental colleges in the Middle East to provide a more comprehensive insight into future directions for this area.

## 5. Conclusions

The majority of the participants in the study demonstrated proper knowledge, positive attitude, and appropriate practice in different aspects of MID. However, the use of magnification tools, such as loupes, still requires more extensive orientation for wider implementation. Additionally, undergraduate dental students tended to choose fluoride for remineralization, while interns were more proficient in MID practices compared to students.

The study found that interns had a higher level of knowledge, attitude, and practice in MID compared to undergraduate students. To achieve better knowledge, attitude, and practices that can promote more conservative clinical practices, it is essential to provide more education and hands-on training on MID concepts during the college curriculum.

## Figures and Tables

**Table 1:** Respondents’ distribution according to gender and academic year level of the dental students (*n* = 163).

Study Data	*n* (%)
Students’ Academic year level Undergraduate:	
Third year	43 (26.4%)
Fourth year	34 (20.9%)
Fifth year	42 (25.8%)
Intern	44 (27.0%)
Gender	
Male	83 (50.9%)
Female	80 (49.1%)

**Table 2:** Respondents’ responses to questions related to assessment of knowledge (K) of MID.

Knowledge Statement	*n* (%)
K1. Do you agree dietary habits should be assessed in all patients?	
Strongly agree	99 (60.7%)
Agree	53 (32.5%)
Uncertain	07 (04.3%)
Disagree	04 (02.5%)
Strongly disagree	00 (00.0%)
K2. Do you agree caries risk assessment should be conducted with all patients?	
Strongly agree	70 (42.9%)
Agree	77 (47.2%)
Uncertain	10 (06.1%)
Disagree	02 (01.2%)
Strongly disagree	04 (02.5%)
K3. Do you agree conservative cavity designs like tunnel and box preparations are effective?	
Strongly agree	60 (36.8%)
Agree	73 (44.8%)
Uncertain	15 (09.2%)
Disagree	12 (07.4%)
Strongly disagree	03 (01.8%)
K4. Do you agree the use of lasers for caries detection should be done frequently?	
Strongly agree	14 (08.6%)
Agree	83 (50.9%)
Uncertain	31(19.0%)
Disagree	16 (9.8%)
Strongly disagree	19 (11.7%)
K5. Do you agree that access opening must be crafted to preserve sound tooth structure?	
Strongly agree	82 (50.3%)
Agree	58 (35.6%)
Uncertain	16 (9.8%)
Disagree	05 (3.1%)
Strongly disagree	02 (1.2%)
K6. Do you agree that Gates Glidden drills should be used routinely during rotary root canal preparation?	
Strongly agree	43 (26.4%)
Agree	68 (41.7%)
Uncertain	32 (19.6%)
Disagree	11 (6.7%)
Strongly disagree	09 (5.5%)
K7. Do you think that in general, large apical sized preparations reduces the bacterial load inside the canal?	
Strongly agree	01 (0.61%)
Agree	09 (5.52%)
Uncertain	71 (43.56%)
Disagree	28 (17.18%)
Strongly disagree	54 (33.13%)
K8. Do you agree that conservative preparation designs should be applied to receive extra coronal restoration?	
Strongly agree	55 (33.74%)
Agree	91 (55.83%)
Uncertain	11 (6.75%)
Disagree	4 (2.45%)
Strongly disagree	2 (1.23%)
K9. Do you agree that the improvement of the Recent adhesive system resulted in increasing the success rate of conservative preparation designs?	
Strongly agree	65 (39.9%)
Agree	86 (52.8%)
Uncertain	7 (04.3%)
Disagree	4 (02.5%)
Strongly disagree	1 (0.6%)

**Table 3:** Respondents’ responses to questions related to assessment of attitude and practice (AP) of MID.

Attitude and Practice Statement	*n* (%)
AP1. Are you planning the use of your restorative materials and techniques according to the patient’s caries risk assessment?	
Never	02 (1.2%)
Sometimes	11 (6.7%)
Often	73 (44.8%)
Always	77 (47.2%)
AP2. Are you familiar with the use of magnification (e.g.,: loupes) for tooth preparation and caries detection?	
Never	97 (59.5%)
Sometimes	15 (9.2%)
Often	19 (11.6%)
Always	32 (19.6%)
AP3. Have you ever used the Chemo-mechanical Cavity Preparation technique in the clinics?	
Never	129 (79.1%)
Sometimes	32 (19.6%)
Often	2 (1.2%)
Always	0 (0.0%)
AP4. Are you depending on the use of radiographs for caries detection?	
Never	03 (01.8%)
Sometimes	52 (31.9%)
Often	46 (28.2%)
Always	62 (38.0%)
AP5. Do you prefer to make large access preparation in general?	
Never	151 (92.64%)
Sometimes	9 (5.52)
Often	3 (1.84%)
Always	0 (0%)
AP6. Do you prefer to use Gates Glidden drills regularly during rotary root canal preparation?	
Never	20 (12.27%)
Sometimes	51 (31.29%)
Often	47 (28.83%)
Always	45 (27.61%)
AP7. Are you practicing the concept of large apical size preparation to reduce the bacterial load in the canal in general?	
Never	55 (33.74%)
Sometimes	91 (55.83%)
Often	13 (7.98%)
Always	4 (2.45%)
AP8. Are you familiar with using recent high strength and high esthetic ceramic materials?	
Never	19 (11.66%)
Sometimes	64 (39.26%)
Often	52 (31.90%)
Always	28 (17.18%)
AP9. Are you familiar with using different bonding systems and techniques for bonding to different ceramic restorations?	
Never	28 (17.18%)
Sometimes	66 (40.49%)
Often	48 (29.45%)
Always	28 (12.88%)

**Table 4:** Comparison between undergraduate students and interns according to their responses to knowledge (K) of MID.

Knowledge of MID		Strongly Agree	Agree	Uncertain	Disagree	Strongly Disagree	c^2^	*p*
K1	Undergraduate (*n* = 119)	58 (48.7%)	50 (42.0%)	7 (5.9%)	4 (3.4%)	0 (0.0%)	27.955 *	^MC^*p* < 0.001 *
	Intern (*n* = 44)	41 (93.2%)	3 (6.8%)	0 (0.0%)	0 (0.0%)	0 (0.0%)		
K2	Undergraduate (*n* = 119)	32 (26.9%)	71 (59.7%)	10 (8.4%)	2 (1.7%)	4 (3.4%)	45.682 *	^MC^*p* < 0.001 *
	Intern (*n* = 44)	38 (86.4%)	6 (13.6%)	0 (0.0%)	0 (0.0%)	0 (0.0%)		
K3	Undergraduate (*n* = 119)	27 (22.7)	64 (53.8%)	13 (10.9%)	12 (10.1%)	3 (2.5%)	36.436 *	^MC^*p* < 0.001 *
	Intern (*n* = 44)	33 (75.0%)	9 (20.5%)	2 (4.5%)	0 (0.0%)	0 (0.0%)		
K4	Undergraduate (*n* = 119)	10 (8.4%)	47 (39.5%)	28 (23.5%)	15 (12.6%)	19 (16.0%)	27.915 *	^MC^*p* < 0.001 *
	Intern (*n* = 44)	4 (9.1%)	36 (81.8%)	3 (6.8%)	1 (2.3%)	0 (0.0%)		
K5	Undergraduate (*n* = 119)	40 (33.6%)	56 (47.1%)	16 (13.4%)	5 (4.2%)	2 (1.7%)	51.923 *	^MC^*p* < 0.001 *
	Intern (*n* = 44)	42 (95.5%)	2 (4.5%)	0 (0.0%)	0 (0.0%)	0 (0.0%)		
K6	Undergraduate (*n* = 119)	8 (7.3%)	59 (53.6%)	32 (29.1%)	11 (10.0%)	0 (0.0%)	83.830 *	<0.001 *
	Intern (*n* = 44)	35 (79.5%)	9 (20.5)	0 (0.0%)	0 (0.0%)	0 (0.0%)		
K7	Undergraduate (*n* = 119)	1 (0.8%)	9 (7.6%)	71 (59.7%)	23 (19.3%)	15 (12.6%)	92.699 *	^MC^*p* < 0.001 *
	Intern (*n* = 44)	0 (0.0%)	0 (0.0%)	0 (0.0%)	5 (11.4%)	39 (88.6%)		
K8	Undergraduate (*n* = 119)	12 (10.1%)	90 (75.6%)	11 (9.2%)	4 (3.4%)	2 (1.7%)	113.418 *	^MC^*p* < 0.001 *
	Intern (*n* = 44)	43 (97.7%)	1 (2.3%)	0 (0.0%)	0 (0.0%)	0 (0.0%)		
K9	Undergraduate (*n* = 119)	21 (17.6%)	86 (72.3%)	7 (5.9%)	4 (3.4%)	1 (0.8%)	99.799 *	^MC^*p* < 0.001 *
	Intern (*n* = 44)	44 (100.0%)	0 (0.0%)	0 (0.0%)	0 (0.0%)	0 (0.0%)		

c^2^: Chi-square test. ^MC^: Monte Carlo. *p*: *p*-value for comparing between the studied groups. *: Statistically significant at *p* ≤ 0.05.

**Table 5:** Comparison of undergraduate students and interns according to their responses for the scoring of their knowledge statement (K).

Knowledge Statement	Undergraduate (*n* = 119)	Intern (*n* = 44)	*t*	*p*
K1	4.36 ± 0.74	4.93 ± 0.25	7.281 *	<0.001 *
K2	4.05 ± 0.85	4.86 ± 0.35	8.648 *	<0.001 *
K3	3.84 ± 0.97	4.70 ± 0.55	7.073 *	<0.001 *
K4	3.12 ± 1.22	3.98 ± 0.51	6.344 *	<0.001 *
K5	4.07 ± 0.89	4.95 ± 0.21	10.135 *	<0.001 *
K6	3.58 ± 0.77	4.80 ± 0.41	12.663 *	<0.001 *
K7	2.65 ± 0.83	1.11 ± 0.32	17.013 *	<0.001 *
K8	3.89 ± 0.69	4.98 ± 0.15	16.243 *	<0.001 *
K9	4.03 ± 0.67	5.0 ± 0.0	15.878 *	<0.001 *

Data are expressed using Mean ± SD. *t*: Student *t*-test. *p*: *p* value for comparing between the studied groups. *: Statistically significant at *p* ≤ 0.05.

**Table 6:** Comparison between undergraduate students and interns according to their responses to attitude and practice (AP) of MID.

Attitude and Practice of MID		Never	Sometimes	Often	Always	c^2^	*p*
AP1	Undergraduate (*n* = 119)	2 (1.7%)	11 (9.2%)	70 (58.8%)	36 (30.3%)	53.840 *	^MC^*p* < 0.001 *
	Intern (*n* = 44)	0 (0.0%)	0 (0.0%)	3 (6.8%)	41 (93.2%)		
AP2	Undergraduate (*n* = 119)	97 (81.5%)	15 (12.6%)	7 (5.9%)	0 (0.0%)	140.566 *	<0.001 *
	Intern (*n* = 44)	0 (0.0%)	0 (0.0%)	12 (27.3%)	32 (72.7%)		
AP3	Undergraduate (*n* = 119)	117 (98.3%)	2 (1.7%)	0 (0.0%)	0 (0.0%)	92.439 *	^MC^*p* < 0.001 *
	Intern (*n* = 44)	12 (27.3%)	30 (68.2%)	2 (4.5%)	0 (0.0%)		
AP4	Undergraduate (*n* = 119)	3 (2.5%)	52 (43.7%)	41 (34.5%)	23 (19.3%)	70.741 *	^MC^*p* < 0.001 *
	Intern (*n* = 44)	0 (0.0%)	0 (0.0%)	5 (11.4%)	39 (88.6%)		
AP5	Undergraduate (*n* = 119)	107 (89.9%)	9 (7.6%)	3 (2.5%)	0 (0.0%)	4.222	0.064
	Intern (*n* = 44)	44 (100.0%)	0 (0.0%)	0 (0.0%)	0 (0.0%)		
AP6	Undergraduate (*n* = 119)	20 (16.8%)	51 (42.9%)	35 (29.4%)	13 (10.9%)	70.746 *	<0.001 *
	Intern (*n* = 44)	0 (0.0%)	0 (0.0%)	12 (27.3%)	32 (72.7%)		
AP7	Undergraduate (*n* = 119)	15 (12.6%)	87 (73.1%)	13 (10.9%)	4 (3.4%)	86.982 *	^MC^*p* < 0.001 *
	Intern (*n* = 44)	40 (90.9%)	4 (9.1%)	0 (0.0%)	0 (0.0%)		
AP8	Undergraduate (*n* = 119)	19 (16.0%)	63 (52.9%)	30 (25.2%)	7 (5.9%)	66.960 *	<0.001 *
	Intern (*n* = 44)	0 (0.0%)	1 (2.3%)	22 (50.0%)	21 (47.7%)		
AP9	Undergraduate (*n* = 119)	28 (23.5%)	66 (55.5%)	25 (21.0%)	0 (0.0%)	102.214 *	<0.001 *
	Intern (*n* = 44)	0 (0.0%)	0 (0.0%)	23 (52.3%)	21 (47.7%)		

c^2^: Chi-square test. ^MC^: Monte Carlo. *p*: *p*-value for comparing between the studied groups. *: Statistically significant at *p* ≤ 0.05.

**Table 7:** Comparison between undergraduate students and interns according to the scoring of their attitude and practice statement (AP).

Attitude and Practice Statement	Undergraduate (*n* = 119)	Intern (*n* = 44)	*t*	*p*
AP1	3.18 ± 0.66	3.93 ± 0.25	10.546 *	<0.001 *
AP2	1.24 ± 0.55	3.73 ± 0.45	26.726 *	<0.001 *
AP3	1.02 ± 0.13	1.77 ± 0.52	9.495 *	<0.001 *
AP4	2.71 ± 0.81	3.89 ± 0.32	13.362 *	<0.001 *
AP5	1.13 ± 0.40	1.00 ± 0.0	3.417 *	0.001 *
AP6	2.34 ± 0.89	3.73 ± 0.45	13.050 *	<0.001 *
AP7	2.05 ± 0.61	1.09 ± 0.29	13.524 *	<0.001 *
AP8	2.21 ± 0.78	3.45 ± 0.55	9.727 *	<0.001 *
AP9	1.97 ± 0.67	3.48 ± 0.51	13.517 *	<0.001 *

Data are expressed using Mean ± SD. t: Student t-test. *p*: *p* value for comparing between the studied groups. *: Statistically significant at *p* ≤ 0.05.

## Data Availability

Data supporting reported results are available upon request through the corresponding author.
